# Multidimensional Machine Learning Personalized Prognostic Model in an Early Invasive Breast Cancer Population-Based Cohort in China: Algorithm Validation Study

**DOI:** 10.2196/19069

**Published:** 2020-11-09

**Authors:** Xiaorong Zhong, Ting Luo, Ling Deng, Pei Liu, Kejia Hu, Donghao Lu, Dan Zheng, Chuanxu Luo, Yuxin Xie, Jiayuan Li, Ping He, Tianjie Pu, Feng Ye, Hong Bu, Bo Fu, Hong Zheng

**Affiliations:** 1 Department of Head, Neck and Mammary Gland Oncology Cancer Center, West China Hospital Sichuan University Chengdu China; 2 Laboratory of Molecular Diagnosis of Cancer Clinical Research Center for Breast, West China Hospital Sichuan University Chengdu China; 3 Big Data Research Center School of Computer Science and Engineering University of Electronic Science and Technology of China Chengdu China; 4 Department of Medical Epidemiology & Biostatistics Karolinska Institutet Stockholm Sweden; 5 Department of Epidemiology and Biostatistics West China School of Public Health Sichuan University Chengdu China; 6 Laboratory of Pathology West China Hospital Sichuan University Chengdu China

**Keywords:** breast cancer, prognosis, machine learning, prediction model

## Abstract

**Background:**

Current online prognostic prediction models for breast cancer, such as Adjuvant! Online and PREDICT, are based on specific populations. They have been well validated and widely used in the United States and Western Europe; however, several validation attempts in non-European countries have revealed suboptimal predictions.

**Objective:**

We aimed to develop an advanced breast cancer prognosis model for disease progression, cancer-specific mortality, and all-cause mortality by integrating tumor, demographic, and treatment characteristics from a large breast cancer cohort in China.

**Methods:**

This study was approved by the Clinical Test and Biomedical Ethics Committee of West China Hospital, Sichuan University on May 17, 2012. Data collection for this project was started in May 2017 and ended in March 2019. Data on 5293 women diagnosed with stage I to III invasive breast cancer between 2000 and 2013 were collected. Disease progression, cancer-specific mortality, all-cause mortality, and the likelihood of disease progression or death within a 5-year period were predicted. Extreme gradient boosting was used to develop the prediction model. Model performance was assessed by calculating the area under the receiver operating characteristic curve (AUROC), and the model was calibrated and compared with PREDICT.

**Results:**

The training, test, and validation sets comprised 3276 (499 progressions, 202 breast cancer-specific deaths, and 261 all-cause deaths within 5-year follow-up), 1405 (211 progressions, 94 breast cancer-specific deaths, and 129 all-cause deaths), and 612 (109 progressions, 33 breast cancer-specific deaths, and 37 all-cause deaths) women, respectively. The AUROC values for disease progression, cancer-specific mortality, and all-cause mortality were 0.76, 0.88, and 0.82 for training set; 0.79, 0.80, and 0.83 for the test set; and 0.79, 0.84, and 0.88 for the validation set, respectively. Calibration analysis demonstrated good agreement between predicted and observed events within 5 years. Comparable AUROC and calibration results were confirmed in different age, residence status, and receptor status subgroups. Compared with PREDICT, our model showed similar AUROC and improved calibration values.

**Conclusions:**

Our prognostic model exhibits high discrimination and good calibration. It may facilitate prognosis prediction and clinical decision making for patients with breast cancer in China.

## Introduction

Breast cancer is a heterogeneous disease with different prognoses. Traditional prognostic factors include tumor size, number of positive lymph nodes, tumor grade, and molecular biomarkers such as estrogen receptor (ER), progesterone receptor (PR), human epidermal growth factor receptor 2 (HER2), and Ki67 [1].

Several prognostic prediction models have recently been developed to assist clinical decision making in breast cancer treatment [2]. These models focused on clinical and pathological factors, as well as gene expression (Oncotype, MammaPrint, BCI, and EndoPredict) [3-8]. Among the prediction models based on clinical and pathological factors, Adjuvant! Online and PREDICT are commonly used [3,4]; however, both of these models are largely based on Caucasian populations, and several validation attempts have revealed suboptimal predictions [2,9-13]. Recently, Wu et al [14] developed a race-specific breast cancer recurrence and survival model but with very few Asians. Therefore, the current models, which are based on specific populations, are inadequate for clinical practice and cannot explain the sizable variability in patient prognosis.

In this study, we aimed to develop a comprehensive prediction model for the prognosis of early invasive breast cancer using machine-learning methods. Our study was based on a large cohort of Chinese patients with breast cancer from West China Hospital, Sichuan University.

## Methods

### Patient Population

Patients records were derived from the Breast Cancer Information Management System (BCIMS) at the West China Hospital of Sichuan University [15]; the cases derived from the BCIMS are representative of breast cancer cases in Southwest China [16]. The BCIMS contains over 16,000 breast cancer patient cases dating back to 1989 and prospectively records patient clinical and pathological characteristics, medical history, diagnosis, laboratory results, and treatments [16].

**Figure 1:**
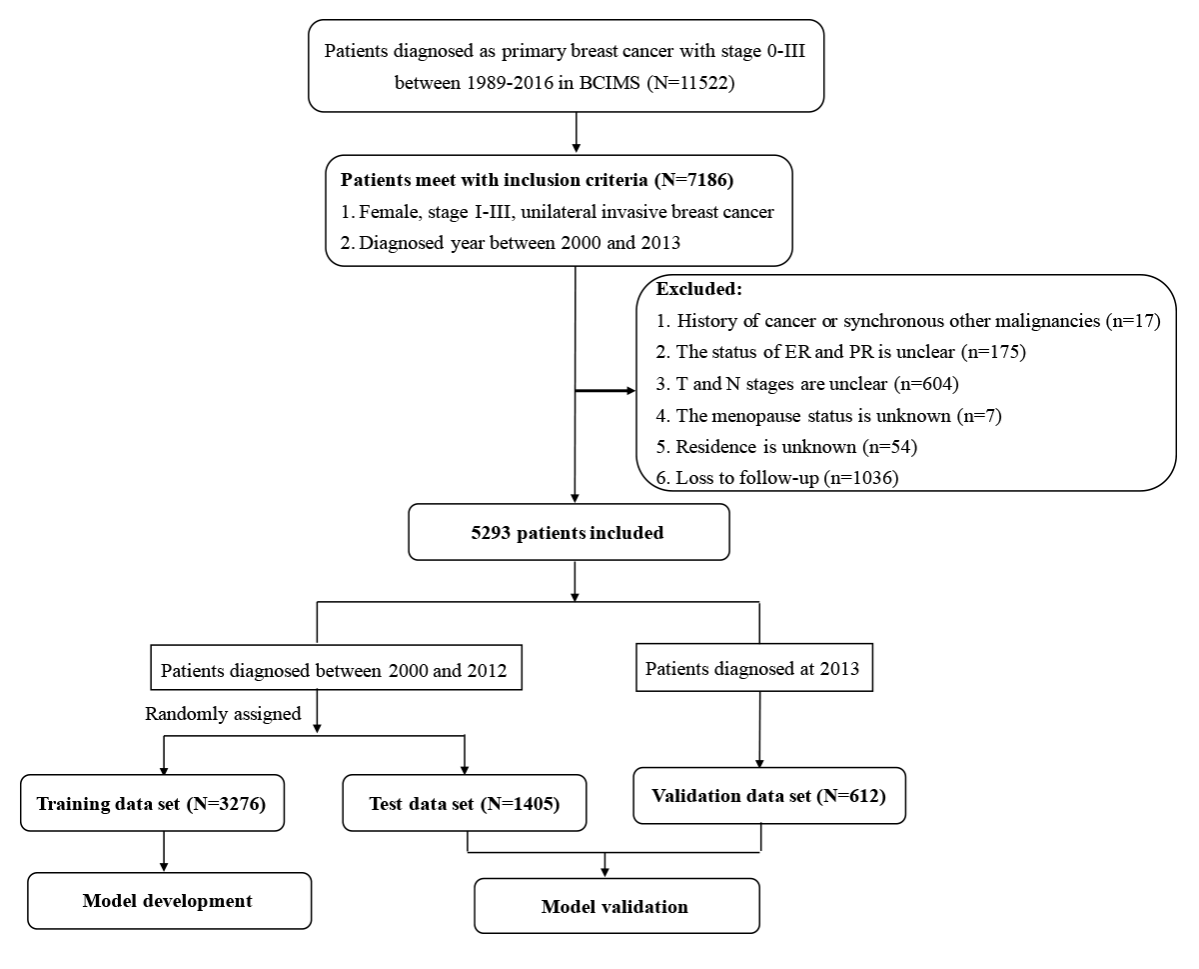
Flowchart of the study design and patient selection. BCIMS: Breast Cancer Information Management System; ER: estrogen receptor; PR: progesterone receptor.

This cohort study included women diagnosed with unilateral stage I to III invasive primary breast cancer who had undergone primary breast cancer treatment between 2000 and 2013. Patients with a history of cancer, with other synchronous malignancies, lacking important information (ER, PR, T stage, N stage, menopause status, and residence), or lost to follow-up were excluded from the study. A flow chart of the study design (with inclusions and exclusions) is shown in Figure 1. In total, 5293 patients were included. Patients diagnosed between 2000 and 2012 were randomly divided into a training set (n=3276) for model development and a test set (n=1405), for model validation, whereas those diagnosed in 2013 were used as a data set (n=612) for model validation in a separate population.

### Outcomes

The patients were prospectively followed using BCIMS records. Follow-up investigations, namely physical examinations, blood tests, breast ultrasounds, computed tomography, and magnetic resonance scans of the chest and abdomen were performed every 3 months for the first 2 years after surgery, then every 6 months from 3 to 5 years after diagnosis, and every year thereafter. Follow-up was conducted via interviews during outpatient visits, or by telephone or postal contact by research assistants.

The endpoints were disease progression (recurrence, metastasis, second primary tumor, and death), cancer-specific mortality (death due to breast cancer), and all-cause mortality. The likelihood of disease progression or death within a 5-year period was predicted. Patients who were alive and showed no evidence of recurrence during the 5 years of follow-up were censored at the fifth year for model development. Invasive disease-free survival was defined as the time from the date of diagnosis to the date of first documented recurrence, the date of death, or 5 years after diagnosis, whichever was earlier. Breast cancer-specific survival was defined as the time from the date of diagnosis to the date of death due to breast cancer or 5 years after diagnosis, whichever was earlier.

### Statistical Analysis

Statistical analyses and modeling were performed using Python (version 3.6.2, Python Software Foundation), XGBoost (version 0.82), and STATA (version 14; Stata Corp LLC) software packages. A chi-square test was used to test the difference in the categorical variables between the training and test data sets. extreme gradient boosting (XGBoost) was used to develop the prognostic prediction model. The process of model development had 2 parts: stratified feature selection and survival modeling. Stratified feature selection has previously been described [17]. Briefly, after setting standards and cleaning the data, 39 original features were obtained to construct prognosis models (Multimedia Appendix 1). Kolmogorov–Smirnov and chi-square tests were preliminarily used to determine whether each feature, as a single factor, was significantly associated with one or more outcomes. This step selected 26 features with notable effects on outcomes. Subsequently, the XGBoost classifier was run to obtain the average importance score of each feature by performing 10-fold cross-validation 5 times with hyperparameter optimization. In this step, the weight method was applied to compute the importance score, which was the number of times a feature was used to split the data across all trees. Subsequently, subsets of features were used to find the threshold score by applying backward selection step-by-step to determine whether a feature score was important. The threshold score was 0.020 for disease progression, 0.015 for cancer-specific mortality, and 0.020 for all-cause mortality. Features with scores lower than the threshold score or with high similarity to other features were excluded. However, menopausal status at diagnosis, which was related to treatment and prognosis in clinical practice, was included, although it scored slightly lower than the threshold. In total, 15 variables were selected for model development (Multimedia Appendix 2). The XGBoost decision tree algorithm was used to estimate the hazard ratio, and hyperparameters were obtained using Bayesian optimization and cross-validation [18]. The likelihood of disease progression or death within a 5-year period was estimated using the equation *ŷ*(*t*, *X*) = 1 – [*S_0_*(*t*)]*^hr^*^(^*^X^*^)^, where, *t* denotes the observed period, *X* denotes the selected variables, *S_0_*(*t*) denotes a population-level baseline survival function, and *hr*() denotes the hazard ratio outputted by the model, respectively. Taking into account the calibration results of the decision tree model, the estimated likelihood was further calibrated using isotonic regression (scikit-learn package, version 0.20.3) [19].

To visualize the contributions of the features in the machine learning model, Shapley additive explanations (SHAP) (shap package, version 0.28.5) and partial dependence plots (PDPbox package, version 0.2.0) were used to evaluate how each feature affected the model prediction. The SHA*P* value represents the effect of changes in a feature on the model output. By pooling the features of all samples in the training data set, the SHA*P* value plot provides an overview of the features that are most important for the model, and features on the plot are sorted by the sum of SHA*P* value magnitudes over all samples [20]. The partial dependence plot takes a row of the data set and repeatedly changes the value for the feature. This is done multiple times with different rows and then aggregated to determine how the feature affects the outcome over a wide range. A partial dependence plot is then created to show how the outcome changes with different values [21].

**Figure 2:**
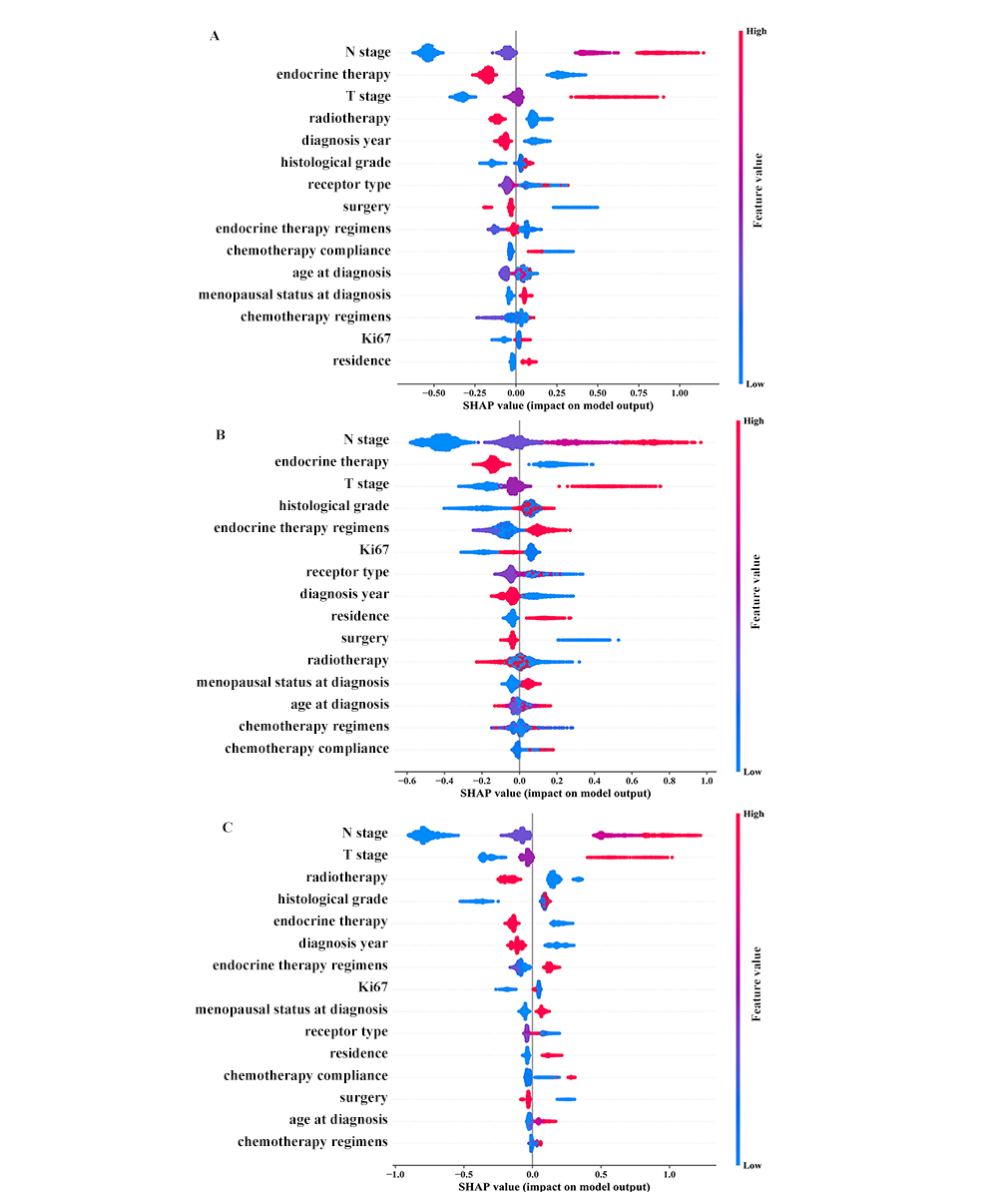
The importance of features for (A) disease progression, (B) breast cancer mortality, and (C) all-cause mortality. SHAP: Shapley additive explanation.

We compared machine learning models incorporating different variables. We also compared the machine learning model with Cox proportional hazards regression models using the same variables. For this purpose, 4 models were developed: (1) a full model with XGBoost incorporating demographic, tumor, and treatment variables (Figure 2 and Multimedia Appendix 3-5); (2) model A with XGBoost incorporating demographic and tumor variables (Multimedia Appendix 6); (3) model B with XGBoost incorporating variables similar to those in other published models (Multimedia Appendix 7, Multimedia Appendix 8) [3,4]; (4) model C with Cox incorporating the same variables as those in the full model (Multimedia Appendix 9).

Model discrimination was evaluated by generating receiver operating characteristic curves and estimating the area under the receiver operating characteristic curves (AUROC) for the models. The DeLong test was used to compare the AUROC values between the models. The predicted and observed 5-year events were compared for each model, and a test of proportion was used for determining the equality between predicted and observed events [14]. A calibration plot was generated using each decile of the predicted value. To explain the different states of breast cancer patients, the model performance was assessed in subgroups of different demographic and tumor characteristics. Our model was also compared with the PREDICT model [4] using test and validation data sets (Multimedia Appendix 7). All statistical tests were 2-sided unless stated otherwise, and a *P* value<.05 was considered statistically significant.

### Ethics

This study was approved by the Clinical Test and Biomedical Ethics Committee of West China Hospital, Sichuan University (reference number 2012-130) on May 17, 2012. Written informed consent was provided by each patient. Data collection started in May 2017 and ended in March 2019. A total of 11,522 patients were recruited, and 5293 patients were included in the analysis for model development and validation.

## Results

### Study Population Characteristics

The training population included 3276 women with a median follow-up period of 7.82 (range 0.01-19.08) years. Of these, 499 women showed disease progression, 202 died from breast cancer, and 261 died from all causes within the first 5 years of follow-up. The test population included 1405 women with a median follow-up period of 8.00 (range 0.01-19.94) years. Of these, 211 women showed disease progression, 94 died from breast cancer, and 129 died from all causes within the first 5 years of follow-up. The validation population included 612 women with a median follow-up period of 5.16 (range 0.01-6.25) years. Of these, 109 women showed disease progression, 33 died from breast cancer, and 37 died from all causes within the first 5 years of follow-up. The demographic, tumor, and treatment characteristics for training, test, and validation data sets are described in Multimedia Appendix 2. The baseline data of patients in the training and test sets were similar, whereas several characteristics differed between training and validation data sets (Multimedia Appendix 2).

### Prognostic Models Incorporating Demographic, Tumor, and Treatment Characteristics

**Figure 3:**
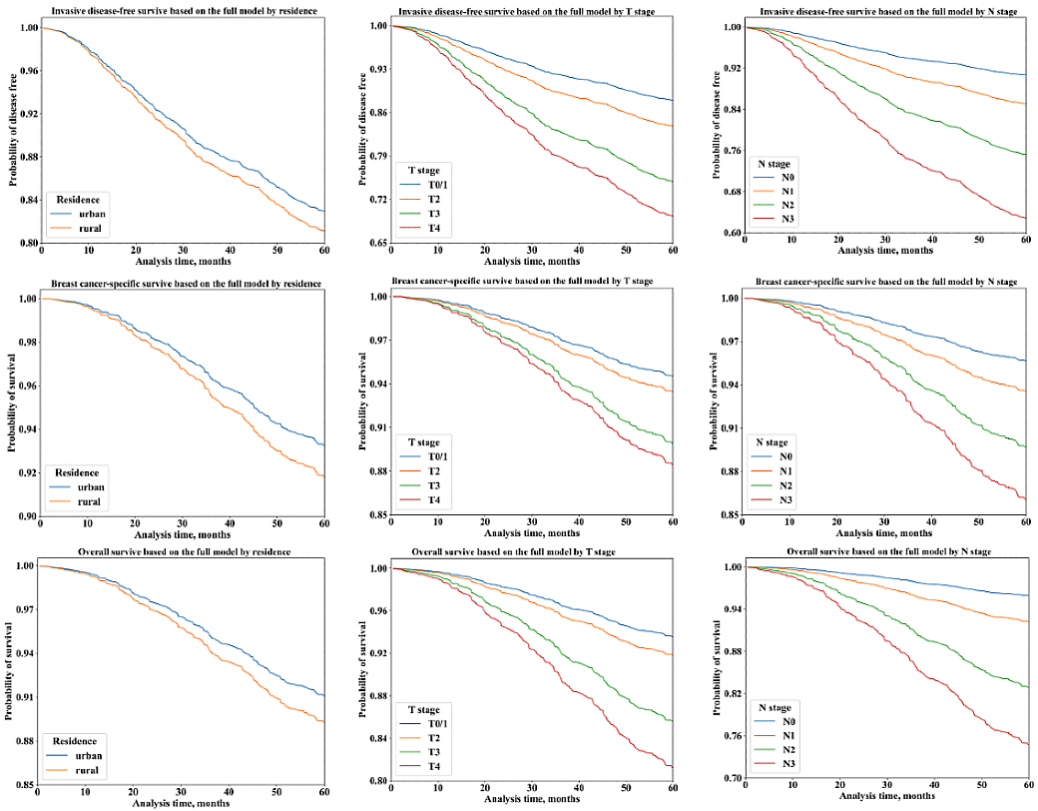
Invasive disease free survival based on the full model (A) by residence, (B) by T stage, and (C) by N stage. Breast cancer–specific survival based on the full model (D) by residence, (E) by T stage, and (F) by N stage. Overall survival based on the full model (G) by residence, (H) by T stage, and (I) by N stage.

Model development used baseline demographic, tumor, and treatment characteristics in the training data set. The full model included age at diagnosis, diagnosis year, menopausal status at diagnosis, residence, T stage, N stage, histological grade, receptor type (ER, PR, HER2), Ki67, surgery, chemotherapy regimens and adherence, radiotherapy, endocrine therapy and regimens. Figure 2 shows variable importance of each outcome according to the SHA*P* value plot. N stage, T stage, endocrine therapy, and radiotherapy ranked as the top features for patient outcomes. The partial dependence plot showed the contribution of a category for each feature (Multimedia Appendix 3-5). The survival curve for the full model based on selected factors is shown in Figure 3.

**Figure 4:**
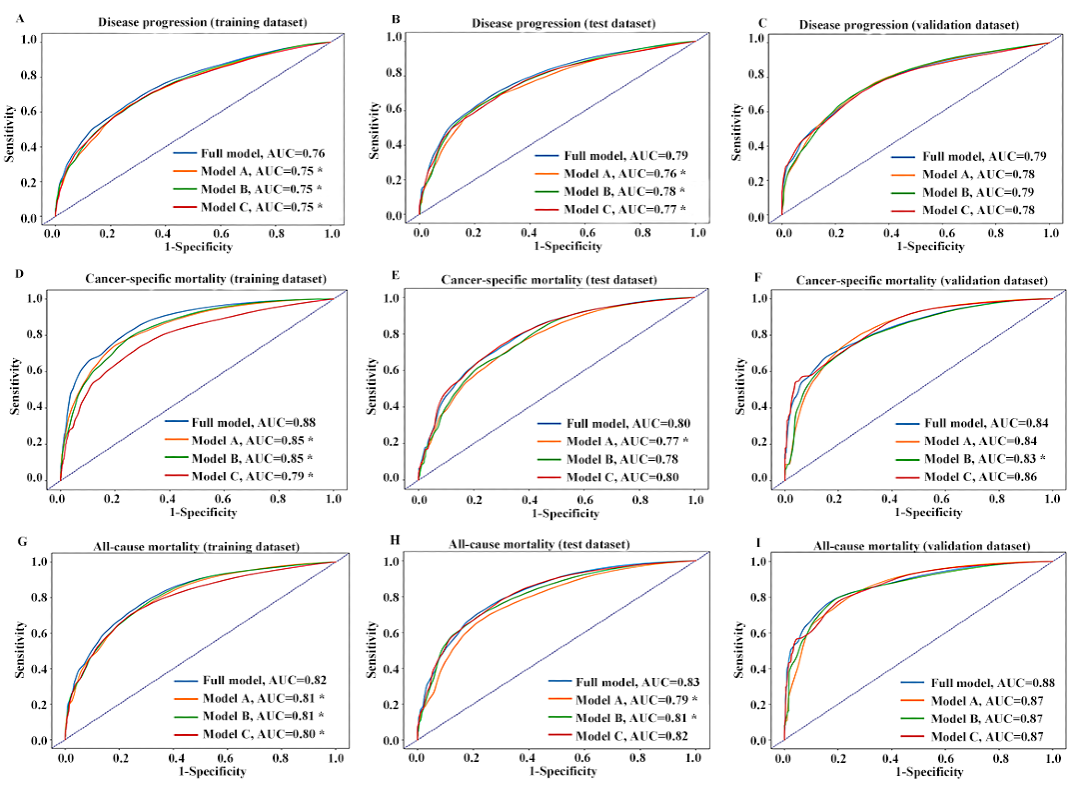
Discriminatory accuracy for predicting breast cancer outcomes: (A) disease progression (training), (B) disease progression (test), (C) disease progression (validation), (D) cancer-specific mortality (training), (E) cancer-specific mortality (test), (F) cancer-specific mortality (validation), (G) all-specific mortality (training), (H) all-specific mortality (test), and (I) all-specific mortality (validation). AUC: area under the curve.

Compared with the other models, the full model exhibited better AUROC with the training data set (disease progression: AUROC 0.76; cancer-specific mortality: AUROC 0.88; all-cause mortality: AUROC 0.82) (Figure 4). The cut-off points were 0.126, 0.064, and 0.072 for disease progression, cancer-specific mortality, and all-cause mortality, respectively. The full model also showed a better AUROC than those of the other models with the test data set (disease progression: AUROC 0.79; cancer-specific mortality: AUROC 0.80; all-cause mortality: AUROC 0.83), except for models B and C for cancer-specific mortality and model C for all-cause mortality (Figure 4). With the validation data set, the full model showed AUROC values comparable with those of the other models (disease progression: AUROC 0.79; cancer-specific mortality: AUROC 0.84; all-cause mortality: AUROC 0.88), except for an improved AUROC for cancer-specific mortality over the AUROC of model B (Figure 4). We also observed good model calibration for each model, except for disease progression prediction with the validation data set (Table 1 and Multimedia Appendix 10).

**Table 1:** Observed and predicted 5-year events.

Data set		Observed	Full model	*P* value	Model A	*P* value	Model B	*P* value	Model C	*P* value
**Disease progression**										
	Test data	211	224.31	.35	221.03	.48	222.29	.43	220.37	.52
	Validation data	109	88.05	.02	89.64	.03	89.16	.03	82.92	.02
**Cancer-specific mortality**										
	Test data	94	98.42	.68	93.51	>.999	94.25	>.999	94.35	>.999
	Validation data	33	36.03	.66	36.98	.55	35.53	.73	34.09	>.999
**All-cause mortality**										
	Test data	129	122.18	.55	119.36	.38	118.03	.31	120.12	.42
	Validation data	37	42.60	.42	43.22	.37	39.54	.74	41.26	.54

### Subgroup Analyses

Discrimination of the full model with the test and validation data sets was evaluated using demographic and tumor characteristics (Table 2). The full model showed good discrimination in most subgroups of the test data set (AUROC 0.70-0.87), except in the ER–/PR–/HER2– and hormone receptor (HR)+/HER2+ subgroups for disease progression and cancer-specific mortality (AUROC 0.63-0.69). With the validation data set, the full model showed good AUROC values for all subgroups (AUROC 0.70-0.97). In addition, the full model was well calibrated in most subgroups of the test data set, except for underestimating the risk of all-cause mortality in the >64-year-old subgroup (*P*=.04) (Table 3). It also showed good calibration in most subgroups of the validation data set, except for underestimating the risk of cancer-specific mortality of ER–/PR–/HER2– patients (4.65 events vs 11 events, *P*=.004) and underestimating the risk of disease progression of the 45 to 54-year-old (25.95 vs 39, *P*=.01), urban (58.91 vs 77, *P*=.01), and HR+/HER2+ (18.34 vs 33, *P*<.001) subgroups.

**Table 2:** AUROC by subgroup analysis.

Subgroup		Test data set, AUROC (95% CI)			Validation data set, AUROC (95% CI)		
		Disease progression	Cancer-specific mortality	All-cause mortality	Disease progression	Cancer-specific mortality	All-cause mortality
**Age at diagnosis**							
	<45 years	0.79 (0.74-0.85)	0.79 (0.71-0.87)	0.83 (0.77-0.89)	0.80 (0.73-0.88)	0.91 (0.81-1.00)	0.94 (0.88-1.00)
	45-54 years	0.80 (0.74-0.86)	0.79 (0.71-0.88)	0.81 (0.74-0.89)	0.79 (0.70-0.88)	0.84 (0.72-0.96)	0.85 (0.73-0.98)
	55-64 years	0.75 (0.67-0.83)	0.80 (0.72-0.88)	0.82 (0.75-0.90)	0.77 (0.63-0.90)	0.80 (0.59-1.00)	0.83 (0.64-1.00)
	>64 years	0.79 (0.67-0.92)	0.82 (0.66-0.97)	0.84 (0.74-0.93)	0.79 (0.65-0.94)	0.80 (0.60-1.00)	0.85 (0.73-0.98)
**Residence**							
	Urban	0.78 (0.73-0.82)	0.80 (0.75-0.86)	0.82 (0.78-0.86)	0.78 (0.74-0.84)	0.84 (0.75-0.93)	0.90 (0.85-0.96)
	Rural	0.81 (0.75-0.87)	0.77 (0.67-0.87)	0.84 (0.77-0.91)	0.80 (0.71-0.90)	0.84 (0.71-0.97)	0.84 (0.70-0.98)
**Receptor type**							
	ER^a^–/PR^b^–/HER2^c^–	0.69 (0.61-0.78)	0.63 (0.52-0.74)	0.70 (0.60-0.79)	0.92 (0.83-1.00)	0.96 (0.92-1.00)	0.97 (0.94-1.00)
	ER–/PR–/HER2+	0.75 (0.63-0.87)	0.86 (0.77-0.96)	0.85 (0.76-0.94)	0.70 (0.53-0.86)	0.87 (0.62-1.00)	0.87 (0.61-1.00)
	HR^d^+/HER2-	0.84 (0.79-0.89)	0.84 (0.78-0.91)	0.87 (0.82-0.92)	0.73 (0.63-0.83)	0.75 (0.59-0.92)	0.83 (0.70-0.97)
	HR+/HER2+	0.69 (0.55-0.82)	0.69 (0.48-0.89)	0.78 (0.63-0.94)	0.87 (0.81-0.94)	0.81 (0.65-0.97)	0.84 (0.70-0.97)

^a^ER: estrogen receptor.

^b^PR: progesterone receptor.

^c^HER2: human epidermal growth factor receptor.

^d^HR: hormone receptor.

**Table 3:** Observed and predicted 5-year events by subgroup analysis.

Data set and subgroup			Disease progression			Cancer-specific mortality			All-cause mortality		
			Observed	Predicted	*P* value	Observed	Predicted	*P* value	Observed	Predicted	*P* value
**Test data set**											
	**Age at diagnosis**										
		<45 years	71	78.61	.38	29	35.36	.31	38	40.09	.79
		45-54 years	73	73.64	.99	27	33.63	.27	41	41.64	.98
		55-64 years	46	52.81	.34	27	21.6	.27	32	29.4	.68
		>64 years	21	19.26	.76	11	7.83	.32	18	11.05	.04
	**Residence**										
		Urban	155	170.96	.20	69	72.75	.69	93	89.95	.78
		Rural	56	53.36	.74	25	25.67	.97	36	32.22	.54
	**Receptor type**										
		ER^a^–/PR^b^–/HER2^c^–	53	56.11	.69	29	24.42	.38	38	32.35	.33
		ER–/PR–/HER2+	25	30.48	.30	10	14.48	.27	15	18.95	.39
		HR+/HER2–	93	102.66	.33	39	43.06	.58	54	53.14	.96
		HR+/ HER2+	23	18.02	.26	8	9.3	.79	12	9.17	.42
**Validation data set**											
	**Age at diagnosis**										
		<45 years	40	31.83	.14	7	12.86	.12	10	13.95	.34
		45-54 years	39	25.95	.01	13	11.72	.81	13	13.38	>.999
		55-64 years	19	22.27	.52	9	8.68	1.00	9	11.41	.55
		>64 years	11	8	.33	4	2.77	.65	5	3.85	.73
	**Residence**										
		Urban	77	58.91	.01	18	23.6	.28	22	27.38	.33
		Rural	32	29.14	.63	15	12.43	.54	15	15.22	>.999
	**Receptor type**										
		ER–/PR–/HER2–	15	10.72	.20	11	4.65	.004	11	6.57	.10
		ER–/PR–/HER2+	15	13	.64	5	5.53	.99	5	6.9	.57
		HR+/HER2–	35	32.02	.64	7	12.92	.12	9	14.3	.19
		HR+/HER2+	33	18.34	<.001	7	7.88	.89	9	9.31	>.999

^a^ER: estrogen receptor.

^b^PR: progesterone receptor.

^c^HER2: human epidermal growth factor receptor.

### Comparison with PREDICT

**Figure 5:**
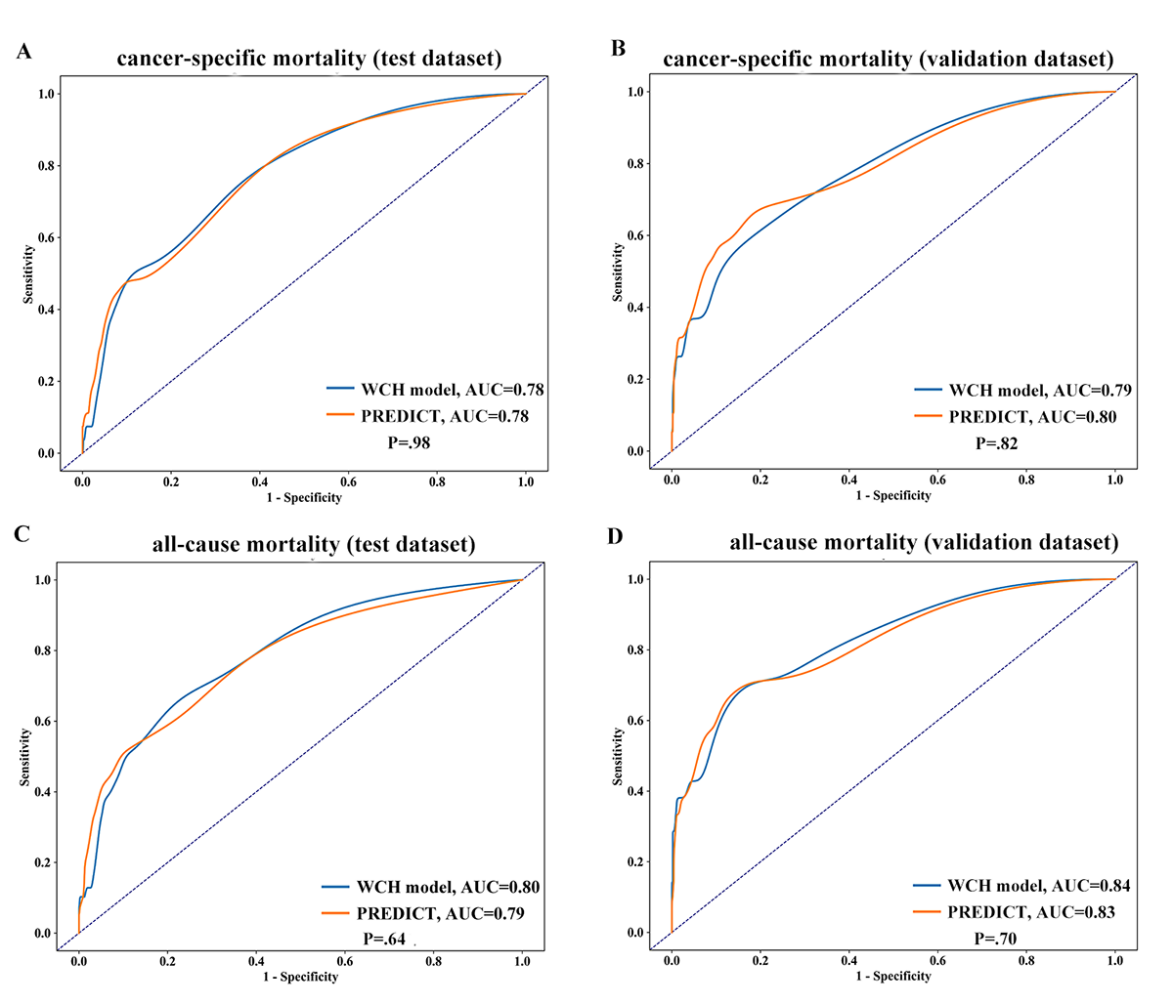
Discriminatory accuracy for (A) cancer-specific mortality (test), (B) cancer-specific mortality (validation), (C) all-specific mortality (test), and (D) all-specific mortality (validation). AUC: area under the curve; WCH: West China Hospital.

We also compared the performance of PREDICT with that of the full model. Both models showed good discrimination and similar AUROC values (0.78-0.84) with the test and validation data sets (Figure 5). However, based on our data, PREDICT was not well calibrated (Table 4). It overestimated the breast cancer specific (80.6 vs 27, 52.6 vs 19, *P*<.001) and all-cause mortalities (93.4 vs 39, 62.1 vs 21, *P*<.001), whereas our model exhibited good calibration.

**Table 4:** Observed and predicted 5-year deaths by full model and PREDICT model.

Calibration		N	Breast cancer–specific mortality			All-cause mortality		
			Observed	Predicted	*P* value	Observed	Predicted	*P* value
**Test data set**								
	PREDICT	602	27	80.6	<.001	39	93.4	<.001
	West China Hospital model	602	27	35.1	.19	39	39.3	>.999
**Validation data set**								
	PREDICT	486	19	52.6	<.001	21	62.1	<.001
	West China Hospital model	486	19	25.2	.24	21	27.5	.24

## Discussion

### Principal Findings

Leveraging the real-world data of 5293 women with primary invasive early breast cancer, we developed a prognostic model to estimate the individual risk of disease progression, cancer-specific mortality, and all-cause mortality using machine learning. Good discriminatory accuracy and calibration were obtained by combining patient demographic, tumor, and treatment factors.

Adjuvant! Online and PREDICT are largely based on Caucasians and have been well validated and widely used in the United States and Western Europe [4,22,23]; however, several validation attempts in non-European countries and even in some European countries revealed suboptimal predictions [2,9-13]. Among the population composition of the race-specific model developed by Wu et al [14], most patients were White, followed by Hispanic and African American, whereas only 518 patients were Asian. In this study, the full model was compared with 3 other models. Compared with model A (demographic and tumor variables) and model B (variables similar to those used in the published models), the full model (demographic, tumor, and treatment variables) exhibited better AUROC, indicating that the additional variables contributed to the improvement in the full model. However, the full model with XGBoost showed AUROC values comparable with those of model C (same variables using Cox proportional hazards regression) in the test and validation data sets, except for a significantly better AUROC for disease progression prediction with the test data set. This showed that the machine learning method, similar to the traditional method, may be suitable for constructing prognostic models based on survival data. There is increasing interest in applying machine learning to clinical data and offering personalized information to support clinical practice [24-27]. Moreover, machine learning provides an innovative approach to data analysis and imaging interpretation, which may be superior to conventional statistics [28]. The ability to automatically handle large multidimensional and multivariate data may ultimately reveal novel associations between specific features and important cancer outcomes. This helps to identify trends and patterns that would otherwise be obscure to investigators [29]. Therefore, a machine learning–based model may play an important role in patient risk stratification [30].

This study also compared the performance of PREDICT with that of our model and showed that the PREDICT algorithm overestimated mortality. This discrepancy is likely due to the lack of data on tumor detection methods [31] as well as to the lack of generalizability to the entire Chinese population. The validation of PREDICT based on an Asian population in another study revealed similar results [9], suggesting that attention should be paid to racial and ethnic differences [32]. Race-specific breast cancer prognosis models for White, Hispanic, and African American patients showed that racial disparity was evident in the distributions of several risk factors and the clinical presentation of the disease [14]. These results suggest that breast cancer prognostic model specific to the characteristics of different populations should be established. To the best of our knowledge, this is the first breast cancer prognosis model based on a Chinese population.

One major merit of our study was the large-scale prospective cohort design with virtually complete follow-up, largely limiting the common sources of bias. Although our study is based on a single institution, the large-scale cohort and complete coverage in West China Hospital guarantee the representativeness of breast cancer patients in Southwestern China. This study is based on real-world data recorded in the BCIMS. The BCIMS infrastructure ensured high quality data collection and virtually complete follow-up through regular interviews, which considerably restricted several common biases such as information and surveillance biases. Several studies have used real-world data to develop cancer models [33-38]. Real-world data are more representative of a patient’s true state than clinical research data.

In real-word practices, some prognostic indicators were missing due to incomplete records of pathological diagnoses in early 2000s, such as histological grade and Ki67 percentage. Some HER2 status data were uncertain because HER2+ results obtained by immunohistochemistry were not further verified by fluorescence in situ hybridization. Although these missing data were inputted as unknown categories in the full model, the model’s good performance relieved this concern to some extent. Moreover, the unknown categories were not related to patient outcome in model C by the Cox method.

The full model incorporated the residential status of breast cancer patients. The incidence of breast cancer in China is generally higher in urban than rural areas, but the associated mortality risk is considerably higher in rural areas [31]. Indeed, the residential status represents the socioeconomic status of Chinese patients to a large extent. Disparities exist between urban and rural patients in terms of lifestyle, medical insurance, ability to afford out-of-pocket treatment expenses, health service, geographical and travel issues, health education, and treatment intention and adherence [39,40]. These factors are associated with patient prognosis [39,41-45]. Moreover, with the progress of urbanization, the residential status of the population is undergoing dynamic changes and should be adjusted in future models.

Our study has some limitations. First, the proposed model showed poor AUROC values (0.63-0.69) for the ER–/PR–/HER2– and HR+/HER2+ subgroups in the test data set. However, it showed good AUROC values for these 2 subgroups in the validation data set (0.81-0.96), which relieves the concern. Notably, this difference in performance between the test and validation data sets was probably because the validation population was diagnosed and treated in 2013, with fewer instances of missing data. Second, the model did not include the variable of targeted therapy. Trastuzumab was approved in China in 2002, but because of its high cost and exclusion from reimbursement in Sichuan province until 2017, the number of HER2+ patients treated with trastuzumab was relatively small in our institution. Third, as a single-center study, our models were developed using a large-scale cohort in the training phase, and the test and validation groups were independent but from the same population. Therefore, validation in an external population is needed in the future.

### Conclusions

We developed and validated a prognostic model for a Chinese population of patients with early-stage invasive breast cancer. Our model showed high discriminatory accuracy and good calibration, which may facilitate prognosis prediction and decision making in clinical practice for Chinese patients with breast cancer.

## Appendix

Multimedia Appendix 1 The candidate features for model development.

Multimedia Appendix 2 Patients characteristics in the training, test, and validation data sets.

Multimedia Appendix 3 The contribution of predictors on disease progression in the full model.

Multimedia Appendix 4 The contribution of predictors on breast cancer mortality in the full model.

Multimedia Appendix 5 The contribution of predictors to all-cause mortality in the full model.

Multimedia Appendix 6 The importance of predictors in model A.

Multimedia Appendix 7 Supplementary methods.

Multimedia Appendix 8 The importance of predictors in model B.

Multimedia Appendix 9 Cox proportional hazards regression model for patient survival.

Multimedia Appendix 10 The calibration plot for each model.

## Abbreviations

AUROC: area under the receiver operating characteristic curve
BCIMS: Breast Cancer Information Management System
ER: estrogen receptor
HR: hormone receptor
HER2: human epidermal growth factor receptor 2
PR: progesterone receptor
SHAP: Shapley additive explanations
XGBoost: extreme gradient boosting
