# Supplementary material for: Multidimensional Machine Learning Personalized Prognostic Model in an Early Invasive Breast Cancer Population-Based Cohort in China: Algorithm Validation Study
Source: JMIR Med Inform. 2020 Nov 9;8(11):e19069. doi: 10.2196/19069 (PMC7683252; doi:10.2196/19069)
Supplement: Multimedia Appendix 1 [file medinform_v8i11e19069_app1.docx]

**Multimedia Appendix 1. The candidate features for model development.**

|  | **Features** |
| --- | --- |
| **Demographic factors** | age at diagnosis |
|  | age at menarche |
|  | diagnosis year |
|  | menopausal status at diagnosis |
|  | age at menopause |
|  | fertility status at diagnosis |
|  | number of pregnancies |
|  | history of lactation |
|  | history of oral contraceptives |
|  | family history of breast cancer |
|  | family history of other cancer |
|  | comorbidity |
|  | with benign breast disease or not |
|  | nationality |
|  | medical insurance |
|  | residence |
| **Tumor characteristics** | histological type |
|  | histological grade |
|  | T stage |
|  | N stage |
|  | clinical/pathological stage |
|  | ER |
|  | PR |
|  | hormone receptor |
|  | HER2 |
|  | molecular type |
|  | Ki67 |
| **Treatment and compliance** | Surgery |
|  | chemotherapy |
|  | neoadjuvant chemotherapy or not |
|  | adjuvant chemotherapy or not |
|  | chemotherapy regimens |
|  | chemotherapy compliance |
|  | radiotherapy |
|  | endocrine therapy |
|  | surgical castration after diagnosis or not |
|  | drug castration after diagnosis or not |
|  | endocrine therapy regimens |
|  | endocrine therapy compliance |
