# Supplementary material for: Multidimensional Machine Learning Personalized Prognostic Model in an Early Invasive Breast Cancer Population-Based Cohort in China: Algorithm Validation Study
Source: JMIR Med Inform. 2020 Nov 9;8(11):e19069. doi: 10.2196/19069 (PMC7683252; doi:10.2196/19069)
Supplement: Multimedia Appendix 2 [file medinform_v8i11e19069_app2.docx]

**Multimedia Appendix 2. Patients characteristics in the training, test, and validation datasets.**

| **Characteristics** | **Training dataset**  **(n=3276)** | | **Test dataset**  **(n=1405)** | | ***P*-value**  **(Training vs**  **Test) *** | | **Validation dataset**  **(n=612)** | | ***P*-value**  **(Training vs**  **Validation)*** |
| --- | --- | --- | --- | --- | --- | --- | --- | --- | --- |
| **Demographic** |  |  | |  | | |  |  | |
| **Age at diagnosis (years)** | | |  | | |  |  | |  |
| <45 | 1203 (36.7%) | | 517 (36.8%) | | | .40 | 234 (38.2%) | | .57 |
| 45-54 | 1173 (35.8%) | | 479 (34.1%) | | |  | 210 (34.3%) | |  |
| 55-64 | 618 (18.9%) | | 293 (20.9%) | | |  | 123 (20.1%) | |  |
| >64 | 282 (8.6%) | | 116 (8.2%) | | |  | 45 (7.4%) | |  |
| **Diagnosis year** |  | |  | | |  |  | |  |
| Before 2008 | 1102 (33.6%) | | 477 (34.0%) | | | .84 | 0 (0) | | <.001 |
| After 2008 | 2174 (66.4%) | | 928 (66.0%) | | |  | 612 (100%) | |  |
| **Menopausal status at diagnosis** | | | | | | | | | |
| Pre-menopause | 1904 (58.1%) | | 810 (57.7%) | | | .77 | 379 (61.9%) | | .08 |
| Post-menopause | 1372 (41.9%) | | 595 (42.3%) | | |  | 233 (38.1%) | |  |
| **Residence** |  | |  | | |  |  | |  |
| urban | 2596 (79.2%) | | 1129 (80.4%) | | | .39 | 433 (70.8%) | | <.001 |
| rural | 680 (20.8%) | | 276 (19.6%) | | |  | 179 (29.2%) | |  |
| **Tumor** |  | |  | | |  |  | |  |
| **T stage ^a)^** |  | |  | | |  |  | |  |
| 0/1 | 1131 (34.5%) | | 479 (34.1%) | | | .78 | 229 (37.4%) | | <.001 |
| 2 | 1807 (55.2%) | | 777 (55.3%) | | |  | 314 (51.2%) | |  |
| 3 | 209 (6.4%) | | 99 (7.0%) | | |  | 25 (4.1%) | |  |
| 4 | 129 (3.9%) | | 50 (3.6%) | | |  | 44 (7.2%) | |  |
| **N stage** |  | |  | | |  |  | |  |
| 0 | 1544 (47.1%) | | 672 (47.8%) | | | .58 | 272 (44.4%) | | .67 |
| 1 | 952 (29.1%) | | 394 (28.0%) | | |  | 189 (30.9%) | |  |
| 2 | 417 (12.7%) | | 168 (12.0%) | | |  | 82 (13.4%) | |  |
| 3 | 363 (11.1%) | | 171 (12.2%) | | |  | 69 (11.3%) | |  |
| **Histological Grade** |  | |  | | |  |  | |  |
| 1 | 92 (2.8%) | | 48 (3.4%) | | | .73 | 17 (2.8%) | | <.001 |
| 2 | 771 (23.5%) | | 325 (23.1%) | | |  | 232 (37.9%) | |  |
| 3 | 1453 (44.4%) | | 624 (44.4%) | | |  | 306 (50%) | |  |
| unknown | 960 (29.3%) | | 408 (29.0%) | | |  | 57 (9.3%) | |  |
| **Molecular type ^b)^** |  | |  | | |  |  | |  |
| ER-/PR-/HER2- | 578 (17.6%) | | 255 (18.1%) | | | .37 | 57 (9.3%) | | <.001 |
| ER-/PR-/HER2+ | 286 (8.7%) | | 125 (8.9%) | | |  | 64 (10.5%) | |  |
| HR+/HER2- | 1868 (57.0%) | | 788 (56.1%) | | |  | 284 (46.45) | |  |
| HR+/HER2+ | 265 (8.1%) | | 135 (9.6%) | | |  | 112 (18.3%) | |  |
| ER-/PR-/HER2 unknown | 93 (2.8%) | | 38 (2.7%) | | |  | 7 (1.1%) | |  |
| HR+/HER2 unknown | 186 (5.7%) | | 64 (4.6%) | | |  | 88 (14.4%) | |  |
| **Ki67** |  | |  | | |  |  | |  |
| <14% | 774 (23.6%) | | 315 (22.4%) | | | .18 | 102 (16.7%) | | <.001 |
| ≥14% | 2132 (65.1%) | | 951 (67.7%) | | |  | 506 (82.7%) | |  |
| unknown | 370 (11.3%) | | 139 (9.9%) | | |  | 4 (0.7%) | |  |
| **Treatment and compliance** | | | | | | | | | |
| **Surgery** |  | |  | | |  |  | |  |
| radical mastectomy | 227 (6.9%) | | 91 (6.5%) | | | .46 | 36 (5.9%) | | <.001 |
| modified radical mastectomy | 2889 (88.2%) | | 1234 (87.8%) | | |  | 522 (85.3%) | |  |
| breast-conserving surgery | 160 (4.9%) | | 80 (5.7%) | | |  | 54 (8.8%) | |  |
| **Chemotherapy compliance** | | | | | | | | | |
| standard **^c)^** | 2804 (85.6%) | | 1192 (84.8%) | | | .77 | 569 (93.0%) | | <.001 |
| not standard | 287 (8.8%) | | 132 (9.4%) | | |  | 21 (3.4%) | |  |
| no chemotherapy | 185 (5.6%) | | 81 (5.8%) | | |  | 22 (3.6%) | |  |
| **Chemotherapy regimens** | | | | | | | | | |
| anthracycline and taxane | 1509 (46.1%) | | 621 (44.2%) | | | .57 | 379 (61.9%) | | <.001 |
| anthracycline | 947 (28.9%) | | 404 (28.8%) | | |  | 104 (17.0%) | |  |
| taxane | 350 (10.7%) | | 171 (12.2%) | | |  | 104 (17.0%) | |  |
| others | 285 (8.7%) | | 128 (9.1%) | | |  | 3 (0.5%) | |  |
| no chemotherapy | 185 (5.6%) | | 81 (5.8%) | | |  | 22 (3.6%) | |  |
| **Radiotherapy** |  | |  | | |  |  | |  |
| yes | 1220 (37.2%) | | 524 (37.3%) | | | .97 | 205 (33.5%) | | .08 |
| no | 2056 (62.8%) | | 881 (62.7%) | | |  | 407 (66.5%) | |  |
| **Endocrine therapy** |  | |  | | |  |  | |  |
| yes | 2170 (66.2%) | | 918 (65.3%) | | | .57 | 469 (76.6%) | | <.001 |
| no | 1106 (33.8%) | | 487 (34.7%) | | |  | 143 (23.4%) | |  |
| **Endocrine therapy regimens** | | | | | | | | | |
| AI | 584 (17.8%) | | 246 (17.5%) | | | .94 | 130 (21.2%) | | <.001 |
| SERM | 938 (28.6%) | | 387 (27.5%) | | |  | 242 (39.5%) | |  |
| SERM/AI | 382 (11.7%) | | 168 (12.0%) | | |  | 48 (7.8%) | |  |
| OFS+SERM/AI | 266 (8.1%) | | 117 (8.3%) | | |  | 49 (8.0%) | |  |
| no endocrine therapy | 1106 (33.8%) | | 487 (34.7%) | | |  | 143 (23.4%) | |  |
| **Events within five years** | | |  | | |  |  | |  |
| **Disease progression** |  | |  | | |  |  | |  |
| 0 | 2777 (84.8%) | | 1194 (85.0%) | | | .85 | 503 (82.2%) | | .11 |
| 1 | 499 (15.2%) | | 211 (15.0%) | | |  | 109 (17.8%) | |  |
| **cancer-specific mortality ^d)^** | | | | | | | | | |
| 0 | 3028 (93.7%) | | 1297 (93.2%) | | | .52 | 555 (94.4%) | | .64 |
| 1 | 202 (6.3%) | | 94 (6.8%) | | |  | 33 (5.6%) | |  |
| **all-cause mortality ^d)^** |  | |  | | |  |  | |  |
| 0 | 2969 (91.9%) | | 1262 (90.7%) | | | .18 | 551 (93.7%) | | .14 |
| 1 | 261 (8.1%) | | 129 (9.3%) | | |  | 37 (6.3%) | |  |

* Chi-square test was employed for different characteristics across patients in training, test, and validation datasets.

^a)^ A total of 12 patients were occult breast cancer, which the T stage is T0 and the N stage is N1-3.

^b)^ HER2 unknown: the immunohistochemistry (IHC) staining showed ++ but did not confirm by FISH, or the FISH result showed uncertainly.

^c)^ Standard chemotherapy: if the patient has completed all course and dose of chemotherapy prescribed by the doctor according to the treatment guidelines, the patient's chemotherapy is considered to be standard.

^d)^ Some patients lost to follow-up after meeting the disease progression endpoint of follow-up. Therefore, the vital status of these patients was not observed at the end of 5-year. In total, for the outcome of mortality, there were 3230 cases for training, 1391 cases for test, and 588 cases for validation.

ER, Estrogen Receptor; PR, Progesterone Receptor; HR, Hormone receptor; HER2, human epidermal growth factor receptor 2; AI, Aromatase inhibitors; SERM, Selective estrogen receptor modulator; OFS, Ovarian function suppression.
