# Supplementary material for: Multidimensional Machine Learning Personalized Prognostic Model in an Early Invasive Breast Cancer Population-Based Cohort in China: Algorithm Validation Study
Source: JMIR Med Inform. 2020 Nov 9;8(11):e19069. doi: 10.2196/19069 (PMC7683252; doi:10.2196/19069)
Supplement: Multimedia Appendix 4 [file medinform_v8i11e19069_app4.docx]

**
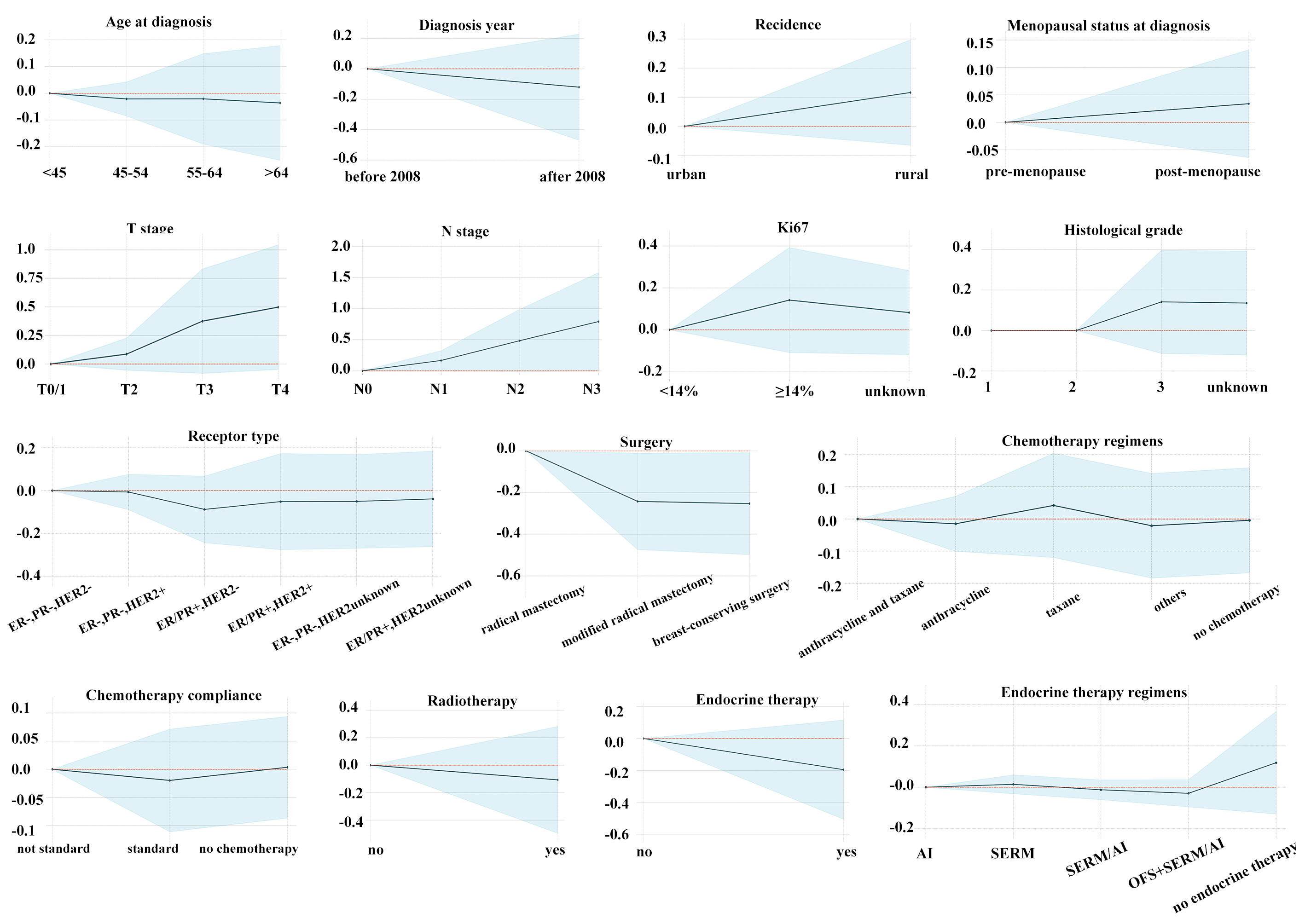
**

**Multimedia Appendix 4. The contribution of predictors on breast cancer mortality in the full model.** The Partial Dependence Plots (PDP) showed how features affect breast cancer mortality. The plot showed the relative probability of breast cancer mortality by each category of a certain feature, in which the first category of the feature was as the reference. The blue area around the line is the line’s confidence.
