# Supplementary material for: Multidimensional Machine Learning Personalized Prognostic Model in an Early Invasive Breast Cancer Population-Based Cohort in China: Algorithm Validation Study
Source: JMIR Med Inform. 2020 Nov 9;8(11):e19069. doi: 10.2196/19069 (PMC7683252; doi:10.2196/19069)
Supplement: Multimedia Appendix 7 [file medinform_v8i11e19069_app7.docx]

**Multimedia Appendix 7. Supplementary Methods**

### Variables for model B

For model B, we chose the variables similar to those in two previous models, namely PREDICT and Adjuvant Online [1,2]. Model B included most of the variables used in these two models except cancer detection mode. The detection mode was not documented in the BCIMS, and no organized program for mammography screening was in place during the study period in Sichuan; therefore, cancer detection mode was not included in the development of model B. In addition, because the BCIMS recorded T and N stages instead of detailed tumor size and positive lymph nodes (especially before 2008), T and N stages were used as proxies of tumor size and positive lymph nodes for model B.

### Comparison with PREDICT

Our model was also compared with the currently available online model PREDICT [2] using the test and validation datasets. Patients who had undergone neoadjuvant chemotherapy or lacked data on histological grade, tumor size, and positive lymph nodes were not included for validation by the PREDICT model. Thus, 604 cases in the test dataset and 490 cases in the validation dataset were applied to the PREDICT model. As mentioned above regarding the cancer detection mode issue, we presumed that the tumors of all of the patients included in our study had been clinically detected.

## References

1. Ravdin PM, Siminoff LA, Davis GJ, Mercer MB, Hewlett J, Gerson N, Parker HL: Computer program to assist in making decisions about adjuvant therapy for women with early breast cancer. *J Clin Oncol* 2001, 19(4):980-991.PMID:11181660

2. Candido Dos Reis FJ, Wishart GC, Dicks EM, Greenberg D, Rashbass J, Schmidt MK, van den Broek AJ, Ellis IO, Green A, Rakha E *et al*: An updated PREDICT breast cancer prognostication and treatment benefit prediction model with independent validation. *Breast Cancer Res* 2017, 19(1):58.PMID:28532503
