# Supplementary material for: Multidimensional Machine Learning Personalized Prognostic Model in an Early Invasive Breast Cancer Population-Based Cohort in China: Algorithm Validation Study
Source: JMIR Med Inform. 2020 Nov 9;8(11):e19069. doi: 10.2196/19069 (PMC7683252; doi:10.2196/19069)
Supplement: Multimedia Appendix 8 [file medinform_v8i11e19069_app8.docx]

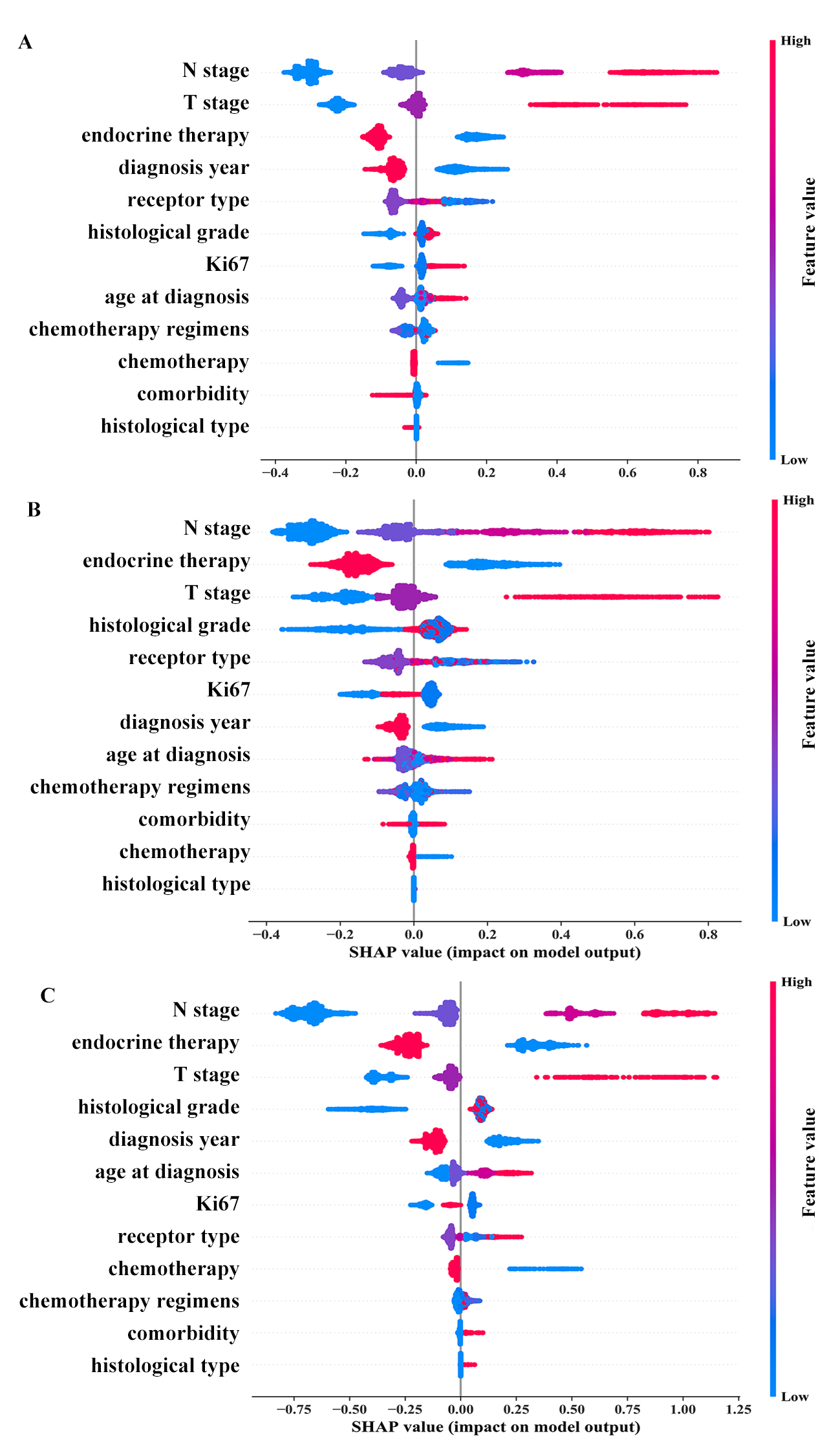
 **Multimedia Appendix 8. The importance of predictors in Model B.** The SHapley Additive exPlanations (SHAP) value plot was used to visualize the importance of features for disease progression (A), breast cancer mortality (B), and all-cause mortality (C). The feature value is the input value for each feature of the model, while the SHAP value represents the effect of changes in a feature on the model output. The horizontal position is the specific effect on the sample outcome, the vertical position shows the feature importance.
