# Supplementary material for: Multidimensional Machine Learning Personalized Prognostic Model in an Early Invasive Breast Cancer Population-Based Cohort in China: Algorithm Validation Study
Source: JMIR Med Inform. 2020 Nov 9;8(11):e19069. doi: 10.2196/19069 (PMC7683252; doi:10.2196/19069)
Supplement: Multimedia Appendix 9 [file medinform_v8i11e19069_app9.docx]

**Multimedia Appendix 9. COX proportional hazards regression model for patient survival.**

| **Variable** | **iDFS**  **Hazard ratio (95%CI)** | **BCSS**  **Hazard ratio (95%CI)** | **OS**  **Hazard ratio (95%CI)** |
| --- | --- | --- | --- |
| **Age at diagnosis** |  |  |  |
| <45 | 1 | 1 | 1 |
| 45-54 | 0.78 (0.61-1.00) | 0.82 (0.55-1.22) | 1.06 (0.75-1.49) |
| 55-64 | 0.85 (0.59-1.23) | 0.76 (0.43-1.35) | 1.08 (0.65-1.81) |
| >64 | 0.80 (0.51-1.25) | 0.74 (0.37-1.51) | 1.21 (0.66-2.20) |
| **Diagnosis year** |  |  |  |
| before 2008 | 1 | 1 | 1 |
| after 2008 | 0.62 (0.49-0.78) | 0.56 (0.39-0.80) | 0.51 (0.37 to 0.70) |
| **Residence** |  |  |  |
| urban | 1 | 1 | 1 |
| rural | 1.13 (0.92-1.40) | 1.45 (1.05-2.00) | 1.35 (1.01 to 1.80) |
| **Menopausal status at diagnosis** |  |  |  |
| pre-menopause | 1 | 1 | 1 |
| post-menopause | 1.24 (0.92-1.66) | 1.37 (0.87-2.14) | 1.05 (0.71 to 1.56) |
| **T stage** |  |  |  |
| T1 | 1 | 1 | 1 |
| T2 | 1.42 (1.12-1.80) | 1.58 (1.04-2.39) | 1.46 (1.03-2.07) |
| T3 | 2.22 (1.60-3.08) | 3.00 (1.79-5.05) | 2.60 (1.66-4.08) |
| T4 | 2.83 (1.99-4.04) | 4.45 (2.58-7.68) | 3.70 (2.31-5.95) |
| **N stage** |  |  |  |
| N0 | 1 | 1 | 1 |
| N1 | 2.00 (1.55-2.58) | 2.75 (1.78-4.24) | 2.63 (1.81-3.83) |
| N2 | 3.75 (2.76-5.09) | 5.39 (3.27-8.90) | 6.14 (3.99-9.43) |
| N3 | 5.46 (4.07-7.31) | 8.31 (5.18-13.33) | 8.76 (5.82-13.18) |
| **Ki67** |  |  |  |
| <14% | 1 | 1 | 1 |
| ≥14% | 1.10 (0.86-1.43) | 1.81 (1.13-2.91) | 1.54 (1.04-2.27) |
| unknown | 1.14 (0.81-1.59) | 1.26 (0.68-2.33) | 1.11 (0.67-1.84) |
| **Histological Grade** |  |  |  |
| 1 | 1 | 1 | 1 |
| 2 | 1.58 (0.57-4.32) | 1.35 (0.18-10.13) | 0.98 (0.23-4.17) |
| 3 | 1.97 (0.72-5.38) | 2.54 (0.35-18.61) | 1.89 (0.46-7.82) |
| unknown | 1.96 (0.72-5.35) | 2.31 (0.31-16.94) | 1.81 (0.44-7.46) |
| **Receptor type** |  |  |  |
| ER-/PR-/HER2- | 1 | 1 | 1 |
| HR+/HER2- | 0.88 (0.66-1.17) | 0.80 (0.51-1.25) | 0.77 (0.52-1.13) |
| ER-/PR-/HER2+ | 1.13 (0.83-1.55) | 1.06 (0.66-1.69) | 1.18 (0.78-1.80) |
| HR+/HER2+ | 1.05 (0.71-1.56) | 0.82 (0.44-1.54) | 0.87 (0.50-1.51) |
| HR+/HER2 unknown | 1.12 (0.73-1.71) | 1.07 (0.54-2.12) | 1.09 (0.61-1.95) |
| ER-/PR-/HER2 unknown | 0.71 (0.41-1.22) | 1.02 (0.50-2.09) | 0.84 (0.43-1.65) |
| **Surgery** |  |  |  |
| radical mastectomy | 1 | 1 | 1 |
| modified radical mastectomy | 0.72 (0.55-0.95) | 0.65 (0.44-0.96) | 0.66 (0.46-0.93) |
| breast-conserving surgery | 0.46 (0.23-0.95) | 0.59 (0.20-1.73) | 0.47 (0.16-1.34) |
| **Chemotherapy compliance** |  |  |  |
| not standard | 1 | 1 | 1 |
| standard | 0.69 (0.51-0.94) | 0.81 (0.49-1.34) | 0.68 (0.44-1.03) |
| no chemotherapy | 0.79 (0.46-1.33) | 0.82 (0.34-1.99) | 1.04 (0.53-2.06) |
| **Chemotherapy regimens** |  |  |  |
| anthracycline and taxane | 1 | 1 | 1 |
| anthracycline | 0.83 (0.64-1.06) | 0.90 (0.60-1.34) | 0.88 (0.62-1.26) |
| taxane | 1.07 (0.77-1.48) | 1.35 (0.83-2.20) | 1.16 (0.75-1.81) |
| others | 0.57 (0.38- 0.86) | 0.68 (0.35-1.32) | 0.62 (0.35-1.10) |
| **Radiotherapy** |  |  |  |
| no | 1 | 1 | 1 |
| yes | 0.62 (0.50-0.77) | 0.52 (0.37-0.72) | 0.45 (0.34-0.61) |
| **Endocrine therapy** |  |  |  |
| no | 1 | 1 | 1 |
| yes | 0.60 (0.42-0.84) | 0.66 (0.38-1.13) | 0.73 (0.46-1.16) |
| **Endocrine therapy regimens** |  |  |  |
| AI | 1 | 1 | 1 |
| SERM | 1.24 (0.88-1.75) | 0.93 (0.54-1.62) | 0.92 (0.57-1.48) |
| SERM/AI | 0.70 (0.44-1.12) | 0.81 (0.40-1.65) | 0.62 (0.33-1.20) |
| OFS+SERM/AI | 0.65 (0.38-1.09) | 0.51 (0.21-1.24) | 0.50 (0.23-1.08) |

iDFS, invasive disease free survival; BCSS, breast cancer specific survival; OS, overall survival; CI, confidence interval; ER, Estrogen Receptor; PR, Progesterone Receptor; HR, Hormone receptor; HER2, human epidermal growth factor receptor 2; AI, Aromatase inhibitors; SERM, Selective estrogen receptor modulator; OFS, Ovarian function suppression.
