# Supplementary material for: Multidimensional Machine Learning Personalized Prognostic Model in an Early Invasive Breast Cancer Population-Based Cohort in China: Algorithm Validation Study
Source: JMIR Med Inform. 2020 Nov 9;8(11):e19069. doi: 10.2196/19069 (PMC7683252; doi:10.2196/19069)
Supplement: Multimedia Appendix 10 [file medinform_v8i11e19069_app10.docx]

**
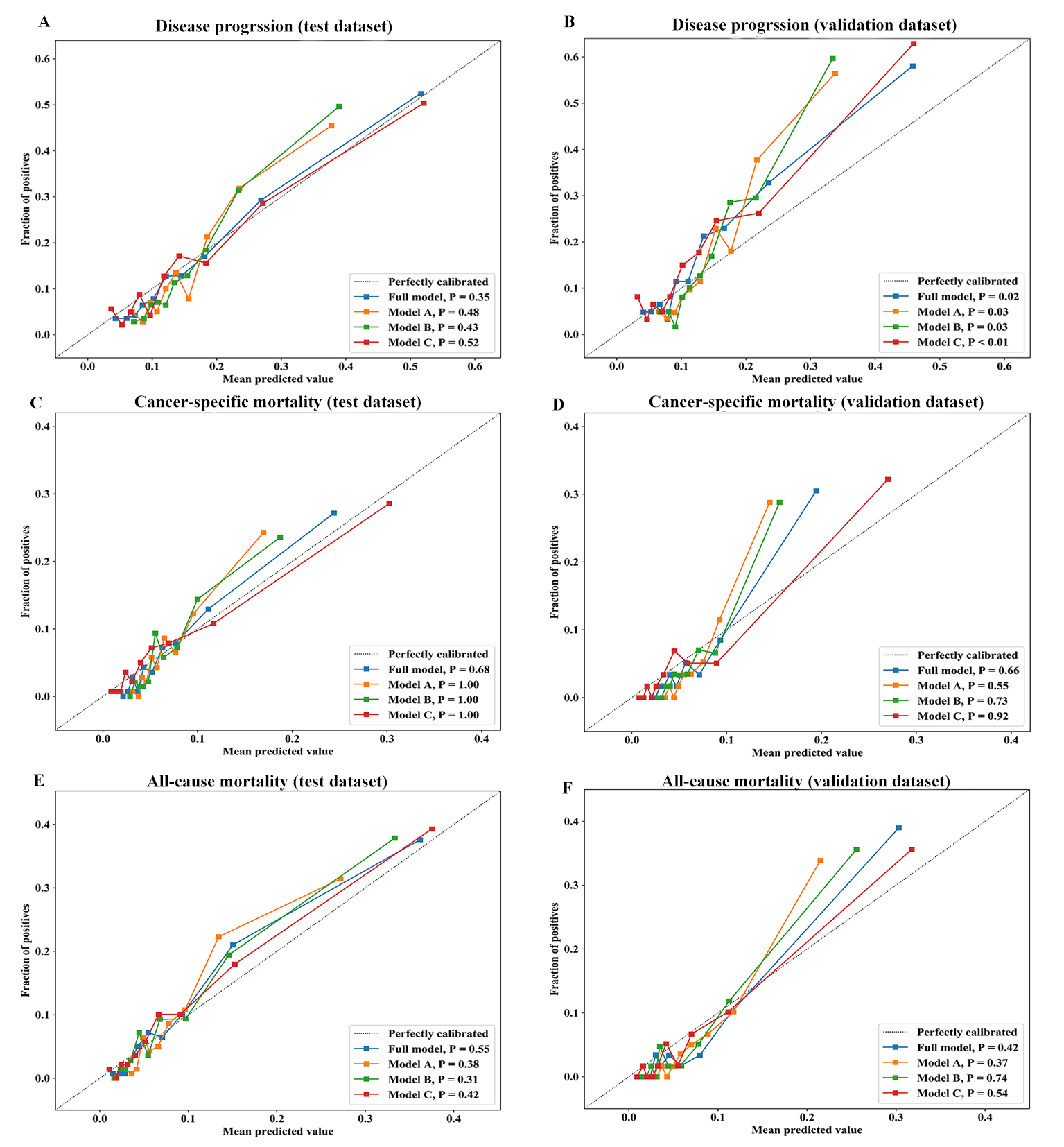
** **Multimedia Appendix 10. The calibration plot for each model.** The calibration plot was generated by the decile of the predicted value. The test of proportion was used to calculate P values.
